# Extract from Foreign Correspondence—The Old University of Bologna

**Published:** 1870-05

**Authors:** 


### EXTRACT FROM FOREIGN CORRESPONDENCE—THE OLD UNIVERSITY OF BOLOGNA.

VENICE, *Feb. 20th, 1870.*

DEAR FATHER:—I left Florence on Thursday last, coming on as far as Bologna, where I stopped over a few hours, in order to visit the *old* and once celebrated University of Bologna; an institution which numbered at one time 4000 students and 300 professors; its medical school, especially, being for a long time the most celebrated in Europe. It was here that the first human body was dissected and the first anatomical prepartions made. The museum is still one of the most complete and extensive in the world, both in human and comparative anatomy.

The library comprises some 15,000 volumes; among them some very valuable manuscripts. The celebrated linguist Mezzofanty, who is said to have spoken fluently 42 different languages.

It is a curious fact, in connection with this university, that several of the most important chairs—those of History, Natural Philosophy, Literature, and even Anatomy, have, at different times, been filled by *ladies*. A wax figure of the lady Anatomist was pointed out to me, in the museum, bending over the table with scalpel in hand, as natural as life, and surrounded on either hand by her own preparations. Another young lady, the daughter of one of the Professors, lectured on history, and is said to have been so beautiful that she was obliged to lecture with a curtain before her face, in order that the attention of the students should not be distracted from the subject of her discourse.

The university is still honorably maintained, there being some 400 students in attendance, I was told. There is quite an extensive hospital in connection with the medical school, where regular clinical instruction is given. I had intended to remain over long enough to attend some of the lectures and clinics, but the weather was very cold and unpleasant, the hotel uncomfortable—no carpets on the floors, and no means of warming any of the rooms—so that I did not think it prudent to remain long, but hastened on here, in hopes of finding pleasanter weather. It is no better here, however—cold, damp, and uncomfortable.

